# Investigation of canine caudal articular process dysplasia of thoracic vertebrae using computed tomography: Prevalence and characteristics

**DOI:** 10.3389/fvets.2023.1066420

**Published:** 2023-02-15

**Authors:** Jiyoung Ban, Jihyeon Park, Hyesung Kim, Kwangyong Yoon, Miju Oh, Yooyoung Lee, Minju Lee, Jinhwa Chang, Byungjin Kim, Jongman Kim, Dongwoo Chang

**Affiliations:** ^1^Section of Veterinary Imaging, Veterinary Medical Center, College of Veterinary Medicine, Chungbuk National University, Cheongju, Republic of Korea; ^2^Korea Animal Medical Center, Cheongju, Republic of Korea; ^3^Bon Animal Medical Center, Suwon, Republic of Korea; ^4^Animal Medical Center Soop, Daejeon, Republic of Korea

**Keywords:** caudal articular process dysplasia, congenital vertebral malformation, computed tomography, magnetic resonance imaging, spinal cord myelopathy

## Abstract

Caudal articular process (CAP) dysplasia is a congenital vertebral malformation that results from the failure of ossification center of articular process located in vertebrae, which includes aplasia or hypoplasia. In previous studies, it was reported to be common in small and chondrodystrophic dogs however, investigated in limited breeds. So we aimed to confirm the prevalence and the characteristics of CAP dysplasia in various breeds, and also to investigate the association of CAP dysplasia and spinal cord myelopathy in neurologically abnormal dogs. In this multicenter, retrospective study, the clinical records and thoracic vertebral column computed tomographic (CT) images of 717 dogs between February 2016 and August 2021 were included and 119 dogs which also underwent magnetic resonance imaging (MRI) examination were evaluated. Overall, 337 of 717 dogs (47.0%) had at least one thoracic CAP dysplasia and the prevalence of CAP dysplasia was significantly higher in dogs with a lower body weight (*P* < 0.0001). A total of 66.4% of toy breeds, 39.0% of small breeds, 20.2% of medium breeds, and 6.0% of large breeds were affected by at least one CAP dysplasia. The most affected vertebra was T4 in toy (48.1%) and small breeds (20.8%), and T5 in medium (20.8%) and large breeds (5.0%). In all groups, prevalence of CAP dysplasia between T1 and T9 was higher than post-diaphragmatic vertebrae (T10–T13). Fifty nine of 119 dogs which underwent both CT and MRI examination had symptoms of spinal cord myelopathy of T3-L3 and twenty-five of 59 dogs (42.3%) had at least one thoracic CAP dysplasia. In that 25 neurologically abnormal dogs, 41 sites of intervertebral disc disease (IVDD) were detected. However, only one dog had both CAP dysplasia and herniated disc at the same level. Also, CAP dysplasia associated non-compressive spinal myelopathy at the same level was found in the other dog. Association CAP dysplasia with spinal myelopathy is speculated but is not confirmed by this study.

## 1. Introduction

The vertebral columns consist of vertebrae, which are connected by the synovial facet (zygapophysial joint) formed by the cranial and caudal articular processes of adjacent vertebrae, arising from the junction of the pedicle and lamina (1). The major role of the cranial and caudal articular processes is to stabilize and restrict spinal motion (2). They provide up to 30% of the stability of the spine (3). The orientation of the articular surfaces of cranial/caudal articular processes at T1–T9 is craniodorsal/caudoventral direction resulting “tiled” orientation (2). Unlike the cranial thoracic vertebrae (T1–T9), the caudal articular surfaces of post-diaphragmatic vertebrae (T10–T13) are medial, facing one another across the midline to form an interlocking network in the sagittal plane, which restricts lateral flexion (2).

Articular process dysplasia is an anomaly affecting the cranial or caudal vertebral articular process, which has been considered congenital in nature (4). Cranial articular process dysplasia is a rare disease, but caudal articular process (CAP) dysplasia is common in small breed dogs (4). CAP dysplasia is an anomaly related to reduced formation or absence of the articular process or facet (5). Abnormal development of the vertebral primary ossification centers results in well-recognized conditions, such as block vertebrae, hemivertebrae, or butterfly vertebrae. However, the underlying pathophysiologic mechanism of vertebral articular process dysplasia was not found yet, which could be a consequence of abnormal development of secondary ossification centers (6). There are few veterinary reports related to CAP dysplasia and has been reported that thoracic vertebral abnormalities, including CAP dysplasia, commonly occur in neurologically normal chondrodystrophic breeds with screw tails such as French bulldogs, English bulldogs, and Pugs (7). The spatial pattern was different between breeds, and the prevalence of CAP dysplasia at post-diaphragmatic vertebrae was higher in Pugs than in other breeds. In another study, CAP dysplasia-associated constrictive myelopathy was observed in the thoracolumbar region and was suspected to be a consequence of chronic micromotion in Pugs (8). CAP dysplasia and intervertebral disc disease (IVDD) were evaluated in an earlier study that used postmortem examination and radiography to assess vertebral column length and intervertebral discs in neurologically normal dogs (4). However, radiography for detecting intervertebral disc herniation is less sensitive than advanced imaging techniques, such as computed tomography (CT) and magnetic resonance imaging (MRI) (6). Therefore, the author questioned the correlation between CAP dysplasia and spinal myelopathy in various breeds.

Radiography can be used to screen for congenital vertebral anomalies, but cross-sectional imaging such as CT and MRI provides detailed information regarding both the bony and soft tissue structures of the spine (2). In toy and small breeds, spinal radiography can underestimate articular process anomalies and degree of CAP dysplasia because of their small size in nature. Recent study had shown that certain MRI sequences are better at evaluating bones than CT, however, as its retrospective nature, when generally evaluating bone anatomy, CT is superior to MRI; therefore, CT was selected to identify any CAP anomalies.

The aims of the current study were as follows: (a) to evaluate the prevalence and anatomical characteristics of thoracic CAP dysplasia in dogs using CT scans and (b) to determine the correlation between CAP anomalies and spinal myelopathy. Therefore, we hypothesized that thoracic CAP dysplasia would be common in toy and small breeds. In addition, breed-specific differences would exist with regard to the prevalence and anatomical locations. Spinal cord myelopathy would be more common in the spine with CAP dysplasia than without CAP dysplasia.

## 2. Materials and methods

### 2.1. Animals

A multicenter, retrospective, cross-sectional study was performed at four veterinary animal medical centers. An electronic medical database of Chungbuk National University Veterinary Teaching Hospital, Bon Animal Medical Center, Korea Animal Medical Center, and Soop Daejeon Animal Medical Center were searched to identify canine patients who underwent CT examination between January 2016 and August 2021, including the complete thoracic vertebral column. Decisions for subject inclusion and exclusion were made by one diagnostic imaging expertise. Dogs were included if medical files and CT images were available for review. Exclusion criteria consisted of dogs with a fracture or lysis of the vertebrae, spinal neoplasm (primary or metastatic), prior spinal surgery history (such as hemilaminectomy), poor image quality which was not available for review and under 1 year of age. And to investigate the association of CAP dysplasia and spinal cord myelopathy, neurologically abnormal dogs that underwent both CT and MRI, had thoracic IVDD with neurological deficits of T3-L3 myelopathy were included. Cases in which IVDD occurred only in the lumbar region were excluded. Data collected for client-owned dogs included signalment (breed, age, sex and body weight), reason for CT studies, neurologic signs, and final diagnosis. Breeds were classified by weight: (i) toy breeds, <5 kg; (ii) small breeds, ≥5 kg and <10 kg; (iii) medium breeds, ≥10 kg and <25 kg, (iv) large breeds, ≥25 kg.

### 2.2. CT and MRI

CT scans were obtained for each dog under general anesthesia and in sternal or dorsal recumbency. The scans included the entire thoracic vertebral column and were performed using one of four different CT scanners: a 4-slice CT scanner (Hi Speed QX/I, GE Medical Co., Milwaukee, WI, USA), a 16-slice scanner (Alexion, Toshiba Medical Systems, Otowara, Japan), a 16-slice CT scanner (SOMATOM scope, Siemens, Tokyo, Japan), and a 64-slice CT scanner (Aquilion 64CFX, Toshiba Medical Systems, Otowara, Japan). CT images were acquired using either an axial or a helical CT unit. The slice thickness and interval in patients was 0.625–2 mm with a pitch of 1.5, using the following parameters: 100–130 mAs tube, 120–140 kV tube voltage, 250–500 mm field of view, 512 × 512 matrix, and a bone reconstruction algorithm. Transverse image reconstruction of the spinal column was performed with 0.625–2 mm slice thickness, and sagittal and dorsal images were reformatted with 0.625–2 mm slice thickness. CT scans were obtained during a state of apnea, achieved by hyperventilation, to minimize motion artifacts.

MRI examinations were performed using one of four scanners: Signa creator 1.5T (GE Healthcare, Milwaukee, WI), Vantage Elan 1.5T (Canon Medical Systems, USA), Vantage Titan 1.5T (Canon Medical Systems, USA), and Airis II 0.3T (Hitachi Medical Systems, Twinsburg, OH, USA). General anesthesia was administered to each dog. Once anesthetized, each patient was positioned in dorsal recumbency, and an MRI examination was performed on the spine. Conventional sequences (T1-weighted, T2-weighted, and T1-weighted with contrast enhancement) were obtained using the transverse, sagittal, and dorsal planes.

### 2.3. Image analysis

CT and MRI imaging data were assessed using a Digital Imaging and Communications in Medicine (DICOM) viewer (Osirix MD 12.0.3; Osirix Pixmeo, Geneva, Switzerland). All evaluations of CT and MRI images were performed independently (i.e., decisions were based on independent opinions), and in random order, by two diagnostic imaging expertises.

**Figure 1:**
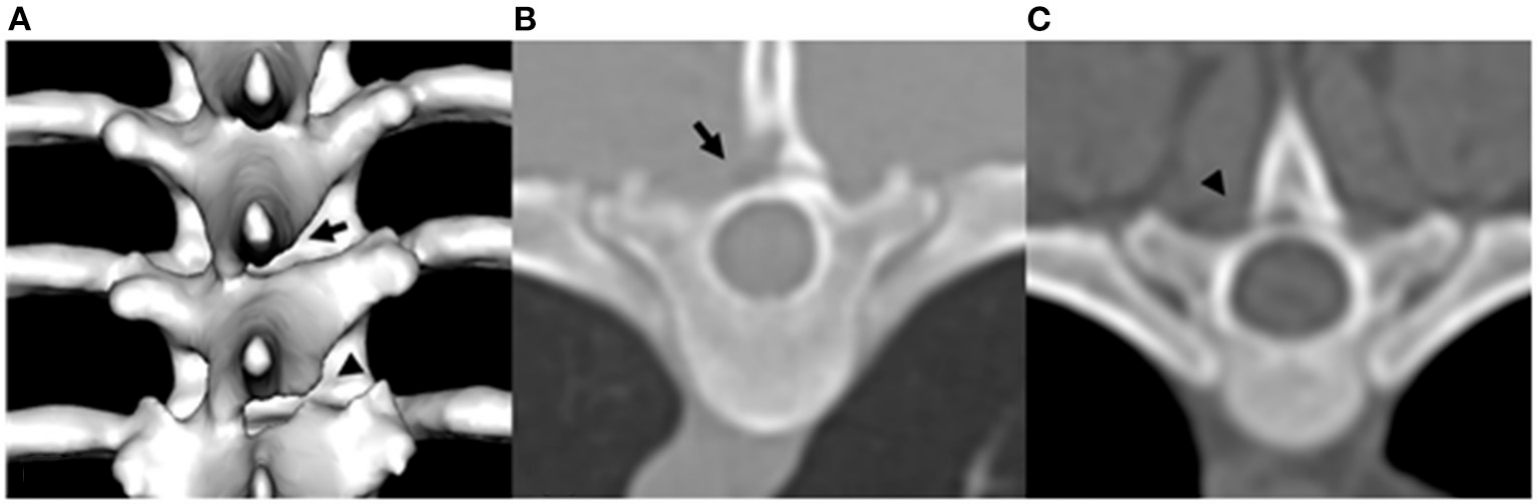
Example of three-dimensional reconstructed CT image of T7 and T8 caudal articular process dysplasia in a Maltese **(A)**, transverse plane image of T6 caudal articular process aplasia in a Poodle **(B)**, and transverse plane image of T7 caudal articular process hypoplasia in a Shih tzu **(C)**. Arrowhead indicates caudal articular process hypoplasia. Arrow indicates caudal articular process aplasia.

CT image analysis of each vertebra was made on transverse, sagittal, dorsal, and three-dimensional reconstructed images using bone reconstruction algorithm with a window width of 1,500 and window level of 300. CAPs were assessed and classified as normal, aplasia, or hypoplasia. CAP dysplasia included hypoplasia and aplasia of the CAP. CAP hypoplasia was defined as the incomplete formation of the CAP. CAP aplasia was defined as the complete absence of the CAP (Figure 1). Each CAP was subjectively classified as normal, hypoplasia, or aplasia. In addition, the occurrence of CAP anomalies was recorded separately on the left and right sides. The number of affected vertebrae was divided into the following types: (1) single, affected only one vertebra, (2) multiple; affected 2–6 vertebrae, (3) generalized, affected vertebrae >6. The occurrence, number, and location of the CAP dysplasia were recorded. After evaluation by two diagnostic imaging expertises, if there was any disagreement, it was reevaluated through consensus. To determine if there was unilateral or bilateral CAP dysplasia at the level of concurrent spinal cord lesions on MRI, and if there were other sites of CAP dysplasia that did not correspond to spinal cord lesions on MRI.

MRI abnormalities of spinal cord myelopathy were made on mid-sagittal T1-weighted, T2-weighted and fluid-attenuated inversion recovery (FLAIR) images. These included intramedullary spinal cord lesions (increase in intra-medullary T2-weighted signal intensity at the affected level), intervertebral disc protrusion (smooth bulging of the annulus fibrosus and dorsal longitudinal ligament to result in loss of epidural fat signal dorsal to the disc and extra-dural spinal cord compression), pre-syrinx or syringomyelia (diffuse poorly marginated increase in T2-weighted signal intensity within the dorsal funiculus of the spinal cord cranial to the affected level, or a cavity containing fluid with signal characteristic similar to cerebrospinal fluid, CSF), and whether spinal cord lesions were apparent at one level or multiple levels.

Spinal cord myelopathy includes both myelopathy associated with intervertebral disc disease, such as acute compressive hydrated nucleus pulposus extrusion, intervertebral disc extrusion, intervertebral disc protrusion, and myelopathy not associated with intervertebral disc disease, which is considered non-compressive spinal cord myelopathy. Acute compressive hydrated nucleus pulposus extrusion was ventral, midline, extradural compressive materials that were hyperintense on T2-weighted sequences at the level of the associated intervertebral disc. Intervertebral disc herniation was defined as the presence of disc material dorsal to the disc space and included both intervertebral disc extrusion and protrusion. Intervertebral disc protrusion was a herniation of disc material and partial loss of the hyperintense signal of the nucleus pulposus on T2-weighted sequence at the same level. Intervertebral disc extrusion was extradural compression of the spinal cord centered over or near the intervertebral disc space. Non-compressive spinal cord myelopathy was considered if there was hyperintensity of the spinal cord on T2-weighted sequence, but not associated IVDD.

### 2.4. Statistical analysis

Patient information was recorded using a Microsoft Excel spreadsheet (Excel for Mac, version 16.54, Microsoft Corporation, Redmond, WA, USA). The mean, SD, and range were used as descriptive statistics. All analyses were performed using GraphPad Prism (GraphPad Prism for Mac OS X, version 9.2.0, GraphPad Software, La Jolla, CA, USA). The difference in the prevalence of CAP anomalies among breeds was assessed using the Chi-square test and Fisher's exact test, corrected for multiple comparisons according to Bonferroni correction. A binary logistic regression was carried out to test age and sex as covariates for the specific-breed prevalence of articular process dysplasia. The Kruskal-Wallis test and Dunn's multiple comparisons test were used to compare the number of affected vertebrae between different breeds. Quantitative data are presented as count numbers or percentages. Statistical significance was set at *p* < 0.05.

## 3. Results

A total of 717 dogs were included in the study. The characteristics (age, body weight and sex) and breed distribution of the included dogs are summarized in Table 1. Toy (316/717; 44.1%) and small breeds (269/717; 37.5%) were overrepresented in included population. There were no significant differences in sex and ages between groups. The reasons indicating the need for a CT scan were metastasis (*n* = 438), neurologic problems (*n* = 156), respiratory disease (*n* = 25), gastrointestinal disease (*n* = 18), congenital vascular disease, such as portosystemic shunt (*n* = 16), health examination (*n* = 15), ear disease (*n* = 11), hepatobiliary disease (*n* = 10), trauma (*n* = 10), urinary tract problems (*n* = 10), oral disease (*n* = 4), and diaphragmatic disorder (*n* = 4).

**Table 1:** The characteristics and breed distribution of 717 dogs underwent CT examination.

	**Dogs which underwent CT examination (*n* = 717)**
Age (years)	9.30 ± 3.33
Body weight (kg)	7.55 ± 7.09
Sex (*n*)	Castrated male (259), Intact male (50), Spayed female (245), Intact female (163)
Breed (*n*)	Maltese (167), Poodle (86), Shih Tzu (83), Mixed (61), Cocker spaniel (39), Pomeranian (39), Yorkshire Terrier (38), Chihuahua (18), Spitz (15), Miniature Pinscher (13), Jindo dog (13), Beagle (12), French bulldog (12), Pekingese (11), Golden Retriever (10), Miniature Schnauzer (8), Welsh corgis (8), Labrador Retriever (7), Bichon Frise (6), Schnauzer (5), Boston terrier (3), Pug (3), Bedllington Terrier (2), Jack Russel Terrier (2), Lhasa Apso (2), Old English sheep dog (2), Pungsan (2), Retriever (2), Samoyed (2), Siberian Husky (2), Standard poodle (2), West Highland White Terrier (2), Alaskan malamute (1), Border collie (1), Bulldog (1), Chowchow(1), Doberman Pinscher (1), Japanese chin (1), Papillon (1), Pointer (1), Scottish Terrier (1), Shiba inu (1), Soft coated wheaten terrier (1), Whippet (1)

Age and body weight represent mean ± standard deviation.

### 3.1. Prevalence and characteristics of caudal articular process dysplasia

**Figure 2:**
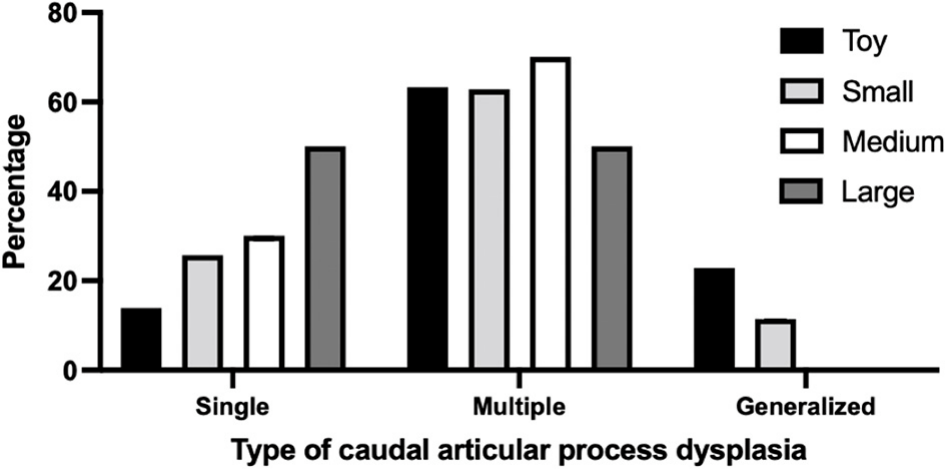
Percentage of type of caudal articular process dysplasia between breeds classified by weight. The number of affected vertebrae was divided into the following CAP dysplasia types: Single, affected only one vertebra; Multiple, affected 2–6 vertebrae; Generalized, affected vertebrae >6. Breeds were classified by weight: (i) toy breeds, <5 kg; (ii) small breeds; 10 kg; (iii) medium breeds; 10–25 kg, (iv) large breeds, >25 kg.

Overall, 337 of 717 dogs (47.0%) had at least one thoracic CAP dysplasia. The toy breeds had the highest prevalence of CAP dysplasia (210/316; 66.4%), followed by small (105/256; 39.0%), medium (20/99; 20.2%) and large breeds (2.33; 6.0%). And the numbers of affected vertebrae according to breed types classified by weight are listed and significant differences are indicated in Table 2. More specifically, 48 of 210 (22.8%) toy breeds and 12 of 105 (11.4%) small dog breeds had the generalized type (>6 vertebrae) of CAP dysplasia (Figure 2). There was no generalized type in the medium and large breeds. In the multiple types, there was no significant difference in prevalence between the breeds. The prevalence of the single type was higher in medium and large breeds than in toy and small breeds.

**Table 2:** Prevalence of caudal articular process dysplasia in 717 dogs according to breed types classified by weight.

**Breeds**	**Number of dogs**	**Number of CAPD (%)**
Toy (<5kg)	316	210 (66.4%)^1, 2, 3^
Small (≥5 kg and <10 kg)	256	105 (39.0%)^1, 4, 5^
Medium (≥10 kg and <25 kg)	99	20 (20.2%)^2, 4^
Large (≥25 kg)	33	2 (6.0%)^3, 5^
Total	717	337 (47.0%)

CAPD, caudal articular process dysplasia. Superscript asterisk indicates a statistically significant difference between two breeds (toy vs. small breeds, 1*P* < 0.0001; toy vs. medium breeds, 2*P* < 0.0001, toy vs. large breeds, 3*P* < 0.0001; small vs. medium breeds; 4*P* < 0.05, small vs. large breeds, 5*P* < 0.01).

Table 3 demonstrates that the prevalence of CAP dysplasia according to breed types. Pekingese had the highest prevalence of CAP dysplasia (9/11; 81.8%), followed by Chihuahuas (14/18; 77.8%), French bulldogs (9/12; 75.5%), Yorkshire Terriers (28/38; 73.7%), Pomeranians (28/39; 71.8%), Maltese (100/167; 59.9%), Poodles (49/86; 57.0%), and Shih tzus (45/83; 54.2%). Pugs, Chow chows, and Bulldogs all had CAP dysplasia, but this was inaccurate because of the small number of dogs.

**Table 3:** Characteristics and type in 24 breeds with caudal articular process dysplasia.

**Breeds**	**Number of dogs (percent)**	**Mean weight (kg)**	**Sex (*n*)**	**Number of CAPD (percent)**	**Single (%)**	**Multiple (%)**	**Generalized (%)**
Chihuahua	18 (2.5%)	3.47 ± 1.07	M (9), F (9)	14 (77.8%)	0 (0%)	12 (85.7%)	2 (14.2%)
Yorkshire Terrier	38 (5.27%)	3.52 ± 2.98	M (10), F (28)	28 (73.7%)	5 (17.8%)	12 (42.8%)	11 (39.3%)
Maltese	167 (23.2%)	3.68 ± 1.41	M (75), F (92)	100 (59.9%)	14 (14%)	62 (62.0%)	24 (24.0%)
Pomeranian	39 (5.4%)	4.05 ± 1.48	M (15), F (24)	28 (71.8%)	5 (17.8%)	19 (67.8%)	5 (17.8%)
Poodle	86 (11.9%)	5.15 ± 1.86	M (39), F (47)	49 (57%)	9 (18.4%)	32 (65.3%)	8 (16.3%)
Pekingese	11 (1.5%)	5.54 ± 1.09	M (3), F (8)	9 (81.8%)	1 (11.1%)	6 (66.7%)	2 (22.2%)
Bichon Frise	6 (0.8%)	5.67 ± 2.66	M (3), F (3)	2 (33.3%)	0 (0%)	2 (100%)	0 (0%)
Miniature Pinscher	13 (1.8%)	5.82 ± 2.40	M (6), F (7)	6 (46.2%)	3 (50.0%)	3 (50.0%)	0 (0%)
Shih Tzu	83 (11.5%)	5.87 ± 1.40	M (40), F (43)	45 (54.2%)	11 (24.4%)	31 (68.9%)	3 (6.7%)
Miniature Schnauzer	8 (1.1%)	5.98 ± 1.52	M (3), F (5)	1 (12.5%)	0 (0%)	1 (100%)	0 (0%)
Pug	3 (0.4%)	7.53 ± 3.00	M (2), F (1)	3 (100%)	0 (0%)	1 (33.3%)	2 (66.6%)
Mixed	61 (8.5%)	7.72 ± 5.08	M (24), F (37)	20 (32.8%)	3 (15.5%)	14 (70.0%)	3 (15.5%)
Bedlington Terrier	2 (0.3%)	7.80 ± 0.28	M (1), F (1)	1 (50%)	0 (0%)	1 (100%)	0 (0%)
Dachshund	28 (3.9%)	7.85 ± 2.51	M (12), F (16)	4 (14.3%)	2 (50.0%)	2 (50.0%)	0 (0%)
Jack Russel Terrier	2 (0.3%)	9.00 ± 0.71	M (1), F (1)	1 (50%)	1 (100%)	0 (0%)	0 (0%)
Spitz	15 (2.1%)	9.17 ± 2.82	M (7), F (8)	4 (26.7%)	2 (50%)	2 (50%)	0 (0%)
Schnauzer	5 (0.7%)	9.44 ± 2.60	M (3), F (2)	2 (40%)	2 (100%)	0 (0%)	0 (0%)
West Highland White Terrier	2 (0.3%)	9.75 ± 2.33	M (2)	1 (50%)	0 (0%)	1 (100%)	0 (0%)
Cocker spaniel	39 (5.4%)	10.78 ± 2.56	M (18), F (21)	3 (7.7%)	1 (33.3%)	2 (66.6%)	0 (0%)
French bulldog	12 (1.7%)	11.60 ± 2.61	M (8), F (5)	9 (75.0%)	1 (11.5%)	8 (88.9%)	0 (0%)
Boston terrier	3 (0.4%)	12.33 ± 2.93	M (3)	2 (66.7%)	1 (50%)	1 (50%)	0 (0%)
Beagle	12 (1.7%)	15.66 ± 5.05	M (6), F (6)	3 (25%)	2 (66.6%)	1 (33.3%)	0 (0%)
Chow chow	1 (0.1%)	24.6	M (1)	1 (100%)	1 (100%)	0 (0%)	0 (0%)
Bull dog	1 (0.1%)	30	M (1)	1 (100%)	0 (0%)	1 (100%)	0 (0%)

CAPD, caudal articular process dysplasia.

**Figure 3:**
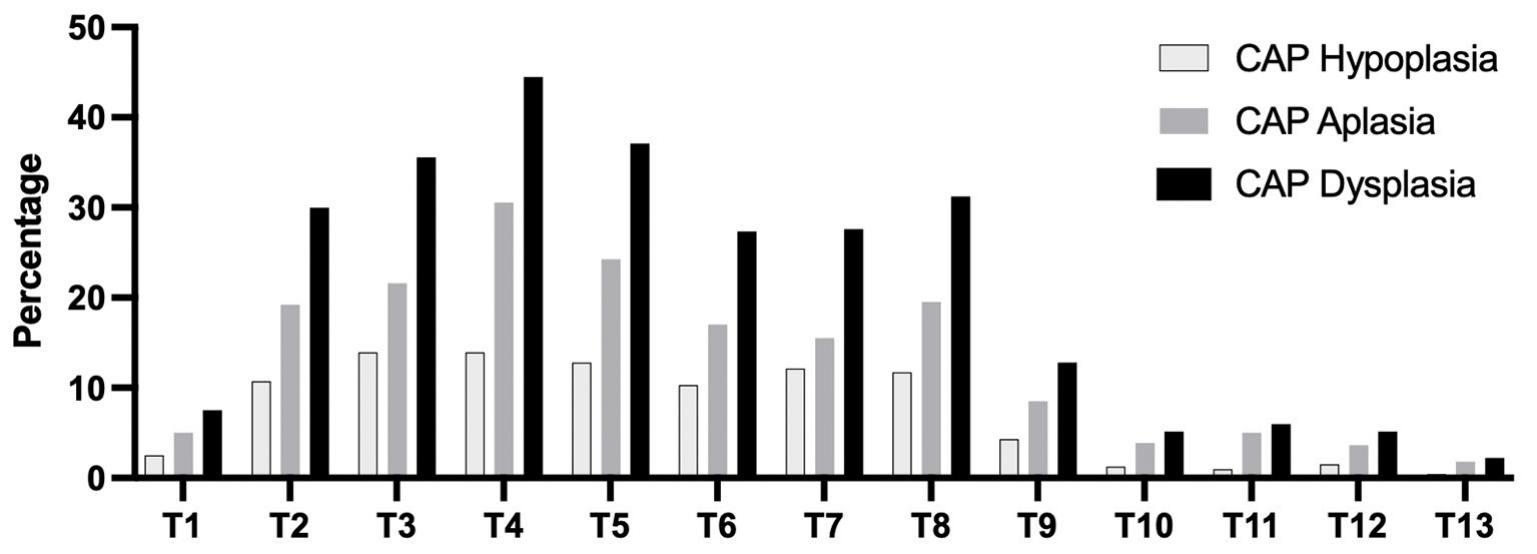
Percentage of anatomic localization of caudal articular process dysplasia, hypoplasia, and aplasia in 717 dogs. CAP, caudal articular process.

**Figure 4:**
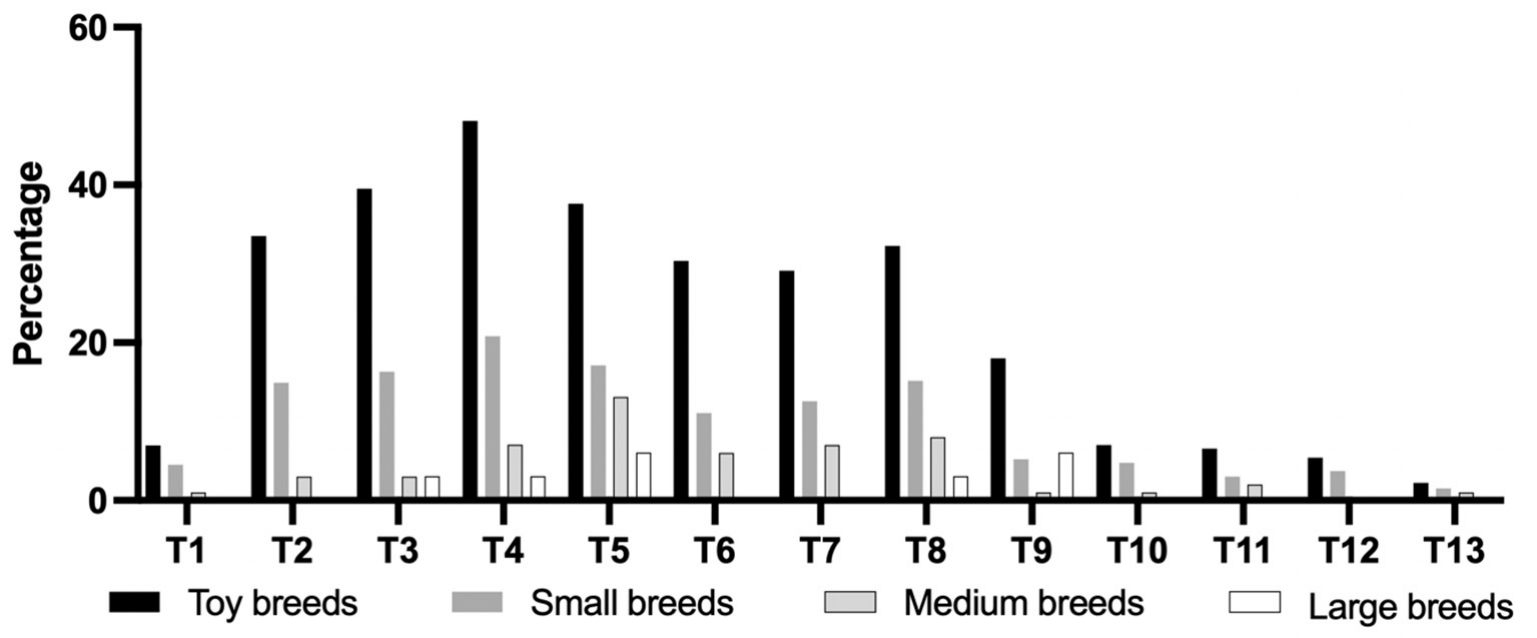
Percentage of anatomic localization of caudal articular process dysplasia between groups according to breed types classified by weight. Toy breeds, <5 kg; Small breeds, ≥5 kg, and <10 kg; Medium breeds, ≥10 kg and <25 kg; Large breeds, ≥25 kg.

Figure 3 shows that the percentage of anatomic localization of thoracic CAP dysplasia in 717 dogs. The most affected vertebra was T4, followed by T5. Figure 4 shows that the percentage of anatomic localization of thoracic CAP dysplasia according to breed types classified by weight. The most affected vertebra was T4 in toy (48.1%) and small breeds (20.8%), and T5 in medium (20.8%) and large breeds (5.0%). In all groups, prevalence of CAP dysplasia between T1 and T9 was higher than post-diaphragmatic vertebrae (T10–T13). The location where the CAP dysplasia occurred was not significantly different. The most frequent region with CAP aplasia was T4 (30.5%). Of the 9,321 vertebrae, 642 (6.8%) showed bilateral CAP dysplasia and 720 (7.7%) showed unilateral CAP dysplasia (533 on the right, 187 on the left). There were no significant differences between right and left sides.

### 3.2. Relationship between caudal articular process dysplasia and spinal cord myelopathy

**Figure 5:**
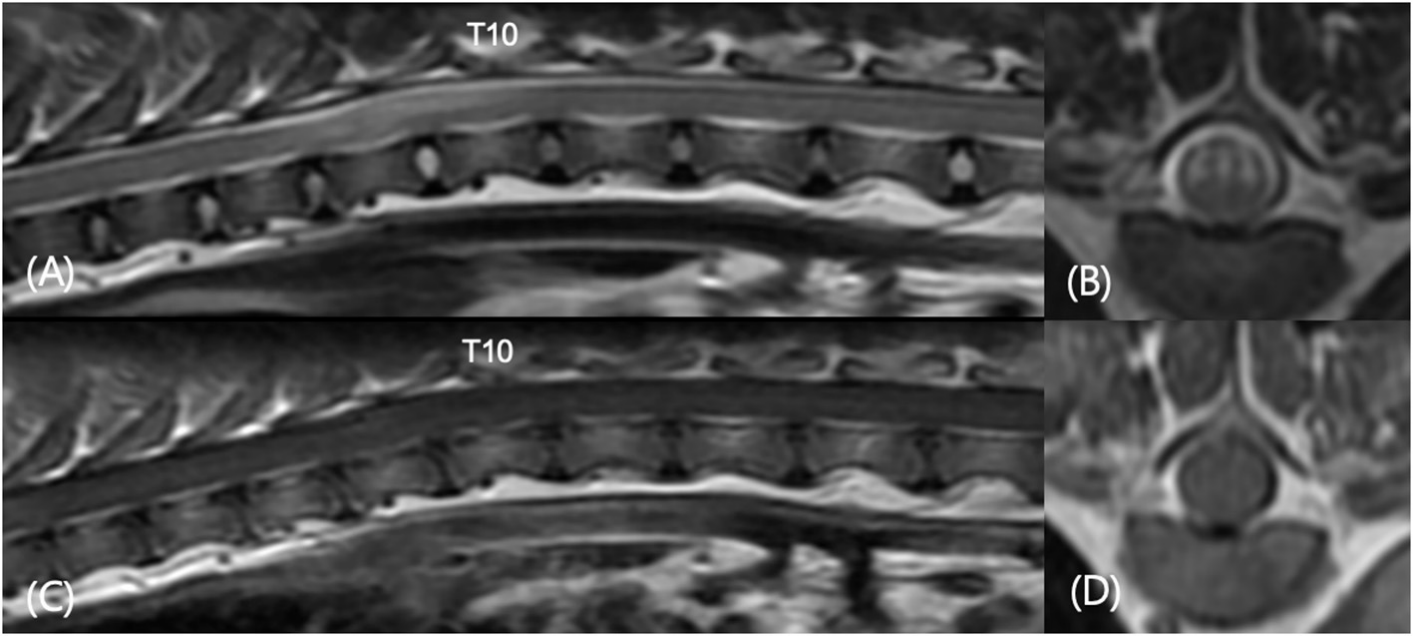
Sagittal T2-weighted **(A)**, transverse T2-weighted T9-T10 **(B)**, sagittal T1-weighted post-contrast **(C)**, and transverse T1-weighted post-contrast T9–T10 **(D)** images in a 3-year-old castrated male in Table 4 (number 8). The patient showed progressive pelvic limb ataxia, urinary retention, and fecal incontinence for 2 years. MRI revealed spinal myelopathy with mild malalignment at T9–T11. No significant findings were found in contrast enhanced images. There were caudal articular process dysplasia at T5, T8, T9, and T11 in CT images.

A total of 119 dogs which underwent both CT and MRI examination were evaluated and 59 of 119 dogs which had symptoms of spinal cord myelopathy of T3-L3 were included. Twenty-five of 59 dogs (42.3%) had thoracic CAP dysplasia. The prevalence of thoracic IVDD in dogs with CAP dysplasia was lower (7.1%) than dogs without CAP dysplasia (8.4%). Table 4 demonstrates the information of 25 dogs with spinal cord myelopathy of T3-L3 and thoracic CAP dysplasia. There were only two cases of CAP dysplasia and IVDD in the same vertebra, and two cases of one vertebra apart. In addition, Figure 5 shows that there was only one dog with non-compressive spinal myelopathy at the same levels. Despite medical treatment, the patient was euthanized at the request of the owner a few months later due to worsening clinical signs.

**Table 4:** Characteristics of 25 dogs with caudal articular process dysplasia and spinal cord myelopathy.

**Signalment**					**Caudal articular process dysplasia**												
**No**.	**Breeds**	**Age**	**Sex**	**Weight**	**T1**	**T2**	**T3**	**T4**	**T5**	**T6**	**T7**	**T8**	**T9**	**T10**	**T11**	**T12**	**T13**
1	Boston terrier	5	CM	13.5					L. h								
2	Chihuahua	5	CM	6.1		R. h		R. h									
3	Chihuahua	8	CM	3.3		R. h	B. h	B. h							R. a		
4	Chihuahua	7	SF	2.9		L. h	B. h	B. h									
5	Cocker Spaniel	8	CM	11				L. h	L. h			R. h					
6	Cocker Spaniel	14	CM	9.1					L. h								
7	French Bulldog	2	CM	9.12					R. a, L. h		B. h		R. a				
8	Maltese	3	CM	4.8					R. a			R. a	R. h		L. h		
9	Maltese	8	CM	4.3		R. h							L. h		L. h		
10	Maltese	12	CM	3.95		R. a	B. a	R. a									
11	Maltese	10	IF	3.5	B. a	R. h, L. a	B. a	B. a	B. a	B. a		R. h					
12	Maltese	15	IF	3.28		B. a	R. a	B. a			L. a	R. h, L. a	R. h, L. a				
13	Maltese	7	CM	3.2		R. a	B. a	R. h								L. h	
14	Maltese	12	SF	3				B. h	B. a	B. a	B. a	B. h	L. h				
15	Maltese	16	CM	2		R. h	R. a	B. a									
16	Maltese	9	CM	6.2							R. h						
17	Maltese	7	IF	2.3				R. h	L. h	R. h		R. a				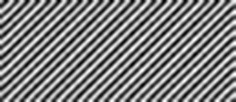	
18	Miniature Pinscher	6	IF	2.5				R. h	L. h	R. h							
19	Pekingese	5	IF	5.5		L. h	B. h										
20	Poodle	11	CM	8.2			B. h										
21	Poodle	14	IF	5.85				R. h			R. h	R. a					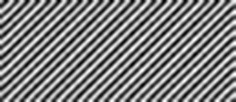
22	Shih Tzu	18	SF	5.35		B. a											
23	Toy poodle	10	CM	3.4							R. a						
24	Yorkshire Terrier	18	CM	4.4		B. a	R. h			L. h		B. a	B. a				
25	Yorkshire Terrier	10	IF	1.65				R. h					R. h				

CM, castrated male; SF, spayed female; IF, intact female. B.a, bilateral aplasia; B.h, bilateral hypoplasia; L.a, left aplasia; L.h, left hypoplasia; R.a, right aplasia; R.h, right hypoplasia; the shadow column indicates 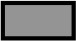, non-compressive spinal cord myelopathy; 
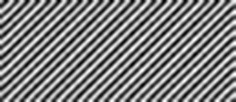
, acute compressive hydrated nucleus pulposus extrusion; 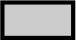, intervertebral disc extrusion; 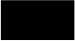, intervertebral disc herniation; 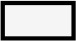, intervertebral disc protrusion.

## 4. Discussion

In this study, 337 of 717 dogs (47.0%) had at least one thoracic CAP dysplasia and the prevalence of CAP dysplasia was significantly higher in dogs with a lower body weight (*P* < 0.0001). A total of 66.4% of toy breeds, 39.0% of small breeds, 20.2% of medium breeds, and 6.0% of large breeds were affected by at least one CAP dysplasia. The most affected vertebra was T4 in toy (48.1%) and small breeds (20.8%), and T5 in medium (20.8%) and large breeds (5.0%). We confirmed that CAP dysplasia would be incidentally found at thoracic vertebrae and there were no clear association between CAP dysplasia and spinal cord myelopathy. To the best of our knowledge, there have been a few studies describing CAP dysplasia and no study has investigated the association between CAP dysplasia and intervertebral disc disease in various breeds (including toy and small breeds) that used CT and MRI. This is the first study to use CT and MRI to describe the correlation between CAP anomalies and intervertebral disc disease by weight. Similar to previous studies, CAP dysplasia was commonly observed in small dogs (toy and small breeds), but it was evaluated in 35 small dog breeds, including only Maltese and Yorkshire terriers (4). In addition, the common site was T8-T7-T9, but in this study, it was confirmed in the T4-T5-T3 order. There were no differences in the anatomical location of the CAP dysplasia between the four weight groups. In all groups, prevalence of CAP dysplasia between T1 and T9 was higher than post-diaphragmatic vertebrae (T10–T13). There were significant differences in the affected number of vertebrae between the groups classified by weight. In total, 48 of 210 (22.8%) toy breeds and 12 of 105 (11.4%) small dog breeds had generalized type of CAP dysplasia. There was no generalized type in the medium and large breeds. The prevalence of the single type was higher in medium and large breeds than in toy and small breeds.

Previous studies had suggested that the prevalence of CAP dysplasia in Pugs was higher in post-diaphragmatic vertebrae (T10–13) compared to English bulldogs and French bulldogs; therefore, it is highly likely that different anatomical distributions rather than numbers themselves affect clinical outcomes. In another study, there was speculation about the causal relationship between the causes of CAP dysplasia and thoracolumbar myelopathy in Pugs and IVDD arachnoid diverticula, and fibrosis were commonly associated with each other (9). However, the prevalence rate is correlated with weight. Lighter weight groups showed higher rates, and heavier weight groups showed lower rates. Table 3 shows that the highest prevalence of CAP dysplasia was in Pekingese (81.8%). This was followed by Chihuahuas (77.8%), French bulldogs (75.5%), Yorkshire Terriers (73.7%), Pomeranians (71.8%), Maltese (59.9%), Poodles (57.0%), and Shih Tzus (54.2%). Pugs, Chow chows, and Bulldogs all had CAP dysplasia, but this was considered to be inaccurate because of the small number of dogs.

Of 717 dogs, one patient showed both CAP anomalies and spinal myelopathy in the same vertebra; however, myelopathy was not associated with intervertebral disc disease. More IVDD was found in the vertebrae without CAP anomalies. The results showed that a higher number of affected thoracic vertebrae did not coincide with a higher prevalence of IVDD. IVDD mainly occurs in the transitional thoracic and lumbar vertebrae, where CAP dysplasia occurs mainly in the anterior 2/3 of the thoracic vertebral column (T1–T9). Intercapital ligaments support the T2–T11 vertebrae and prevent IVDD. The author suggests that the CAP has little effect on vertebral stability in low-weight dogs. It can further be speculated that CAP anomalies alone are less likely to cause spinal myelopathy. In a previous study, due to aplasia or hyperplasia of the thoracolumbar region, bone proliferation was seen with degenerative joint disease, and compressive myelopathy caused by compensatory hyperplasia of the cranial articular facets and protrusion of the ligamentum flavum through the vertebral canal (10). In another study, there were cases of secondary constrictive myelopathy in CAP dysplasia in West highland white terrier dogs (11). Spondylosis deformans were found in 115 of 717 dogs, of which spondylosis deformans and CAP dysplasia occurred in the same vertebra only 16 dogs.

This study was limited by several factors. There are more toy and small breeds than medium and large breeds in South Korea; as such, the number of medium and large breeds in this study is insufficient and may not show an accurate distribution of medium and large breeds. Because of its retrospective design, dogs included in the neurologically normal group did not undergo neurological examination. They were considered neurologically normal when they underwent CT for reasons unrelated to neurological disease and when the medical files did not mention any neurological abnormality. Therefore, it cannot be excluded that these dogs may have had mild neurological abnormalities because of their vertebral articular process anomalies. And the dogs in this group did not undergo MRI. In addition, some dogs may develop clinical signs associated with CAP dysplasia later in life. Only one dog was diagnosed with both CAP dysplasia and spinal cord myelopathy without IVDD. Since CAP anomalies and spinal cord myelopathy were evaluated only when both MRI and CT scans were taken, patients who only underwent MRI were not included. Therefore, there may have been cases in which CAP dysplasia was not evaluated in patients with spinal cord myelopathy. In addition, when dividing patients into groups, only weight was considered, and the body condition score was not assessed. A large cohort study is needed to prove the association between CAP dysplasia and spinal cord myelopathy. Further studies are needed to determine the causal relationship between vertebral bodies, malformations, and thoracic vertebral disc disease in dogs.

In conclusion, the findings supported that toy and small dogs under 10 kg commonly have thoracic CAP dysplasia, but this could be incidental finding. In addition, CAP dysplasia of the cranial thoracic vertebrae is not associated with IVDD, but it is difficult to evaluate the association of CAP dysplasia of the caudal thoracic vertebrae with IVDD are few sample.

## Data availability statement

The raw data supporting the conclusions of this article will be made available by the authors, without undue reservation.

## Ethics statement

Ethical review and approval was not required for the animal study because this is multicenter, retrospective study. Written informed consent was obtained from the owners for the participation of their animals in this study.

## Author contributions

JB, JP, HK, KY, JC, BK, JK, and DC contributed to the case management. JB wrote the first draft of the manuscript. JB, JP, HK, KY, MO, YL, ML, and DC participated in the revision of the manuscript. All authors have read, commented on, and approved the final manuscript.

## References

[1] Evans HE. The skeleton: the vertebral column. In: Evans HE, editor. Miller's Anatomy of the Dog. 3rd edition. Philadelphia: WB Saunders Co., (1993). p. 166–81.

[2] Bouma J. L. Congenital malformations of vertebral articular processes in dogs. Vet Clin North Am Small Anim Pract. (2016) 46:307–26. 10.1016/j.cvsm.2015.10.006 26851714

[3] Hirsch C. The reaction of intervertebral discs to compression forces. J Bone Joint Surg Am. (1955) 37A:1188–96. 10.1016/j.esas.2011.03.001 13271464

[4] Breit S. Osteological and morphometric observations on intervertebral joints in the canine pre-diaphragmatic thoracic spine. (Th1–Th9) Vet J. (2002) 164:216–23. 10.1053/tvjl.2002.0714 12505394

[5] Morgan JP. Congenital anomalies of the vertebral column of the dog: a study of the incidence and significance based on a radiographic and morphologic study 1. Vet Radiol. (1968) 9:21–9. 10.1111/j.1740-8261.1968.tb01082.x

[6] Mai W. Magnetic Resonance Imaging and Computed Tomography Features of Canine and Feline Spinal Cord Disease. Textbook of Veterinary Diagnostic Radiology. St. Louis, MO: Elsevier. (2018). p271-304.

[7] Ryan R Gutierrez-Quintana R ter Haar G De Decker S. Prevalence of thoracic vertebral malformations in French bulldogs, Pugs and English bulldogs with and without associated neurological deficits. Vet J. (2017) 221:25–9. 10.1016/j.tvjl.2017.01.018 28283076

[8] Lourinho F Holdsworth A McConnell JF Gonçalves R Gutierrez-Quintana R Morales C. Clinical features and MRI characteristics of presumptive constrictive myelopathy in 27 pugs. Vet Radiol Ultrasound. (2020) 615:545–54. 10.1111/vru.12890 32583954

[9] Driver CJ Rose J Tauro A Fernandes R Rusbridge C. Magnetic resonance image findings in pug dogs with thoracolumbar myelopathy and concurrent caudal articular process dysplasia. BMC Vet Res. (2019) 15:1–10. 10.1186/s12917-019-1866-0 31151444 PMC6544997

[10] Fisher SC Shores A Simpson ST. Constrictive myelopathy secondary to hypoplasia or aplasia of the thoracolumbar caudal articular processes in Pugs (1993–2009). J Am Vet Med Assoc. (2013) 242:223–9. 10.2460/javma.242.2.223 23276100

[11] Ros C de la Fuente C de Carellán Mateo AG Laborda-Vidal P. Constrictive myelopathy secondary to caudal articular vertebral process dysplasia in West Highland white terrier dogs. Can Vet J. (2020) 61:1155. 33149351 PMC7560773

